# Urinary protein profiling in hyperactive delirium and non-delirium cardiac surgery ICU patients

**DOI:** 10.1186/1477-5956-9-13

**Published:** 2011-03-22

**Authors:** Mark van den Boogaard, Rachel PL van Swelm, Frans GM Russel, Suzanne Heemskerk, Johannes G van der Hoeven, Rosalinde Masereeuw, Peter Pickkers

**Affiliations:** 1 Department of Intensive Care Medicine, Radboud University Nijmegen Medical Centre, P.O. Box 9101, Nijmegen, 6500HB, the Netherlands; 2 Department of Pharmacology and Toxicology, Radboud University Nijmegen Medical Centre, Nijmegen Centre for Molecular Life Sciences, P.O. Box 9101, Nijmegen, 6500HB, the Netherlands; 3 Nijmegen Institute for Infection, Inflammation and Immunity (N4i), Radboud University Nijmegen Medical Centre, P.O. Box 9101, Nijmegen, zip code 6500HB, the Netherlands

## Abstract

**Background:**

Suitable biomarkers associated with the development of delirium are still not known. Urinary proteomics has successfully been applied to identify novel biomarkers associated with various disease states, but its value has not been investigated in delirium patients.

**Results:**

In a prospective explorative study hyperactive delirium patients after cardiac surgery were included for urinary proteomic analyses. Delirium patients were matched with non-delirium patients after cardiac surgery on age, gender, severity of illness score, LOS-ICU, Euro-score, C-reactive protein, renal function and aorta clamping time. Urine was collected within 24 hours after the onset of delirium. Matrix-assisted laser desorption/ionisation-time of flight mass spectrometry (MALDI-TOF MS) was applied to detect differences in the urinary proteome associated with delirium in these ICU patients. We included 10 hyperactive delirium and 10 meticulously matched non-delirium post-cardiac surgery patients. No relevant differences in the urinary excretion of proteins could be observed.

**Conclusions:**

We conclude that MALDI-TOF MS of urine does not reveal a clear hyperactive delirium proteome fingerprint in ICU patients.

**Trial Registration:**

Clinical Trial Register number: NCT00604773

## Background

Delirium is an acute psycho-organic syndrome, that frequently occurs in hospitalized patients and particularly in critically ill patients. This neuropsychiatric disorder is associated with serious health problems, such as prolonged stay on the mechanical ventilator, in the intensive care unit (ICU) and hospital, and a higher mortality rates [1]. Three subtypes of delirium; hyperactive, hypoactive and a mixed subtype, can be distinguished based on patients Richmond Agitation Sedation Scores (RASS) [2]. In daily practice, nurses and physicians experience the most difficulties with the hyperactive delirium patients who are often aggressive or even combative and in whom their delirium is associated with dislocation of their endotracheal tube and other lifesaving materials.

Although the pathophysiology of delirium is far from clear, several biomarkers and pathways, such as neuro-anatomic abnormalities, cholinergic failure, inflammatory responses and activation of the hypothalamic-pituitary adrenal axis, were found to be associated with the development of delirium [3;4]. Nevertheless, suitable biomarkers that may facilitate the diagnosis of delirium have not been discovered.

Proteomics is a profiling method to detect a wide range of markers simultaneously. This technique allows the identification of several proteins potentially involved in the pathophysiological mechanism of disorders [5], such as delirium. Proteomics can be applied for determinations in tissue [6] and in several biological fluids, i.e. cerebro-spinal fluid and serum [7-9]. Differences in protein profiles were detected in brain tissue of hyperactive delirium rats (significant peak at *m/z *5030 and 5179) [10] and in the serum of delirium elderly patients with hip fracture (significant peak at *m/z 15,900 *identified as haemoglobin-β) [8]. Proteomics of urine samples is of special interest, as urine reflects the low molecular weight protein pool of blood without, for mass spectrometry disturbing, abundant proteins, such as albumin [11]. In addition, urine can be collected in a non-invasive way. Proteomics of urine has proven to be useful in predominantly urogenital diseases, but has recently also been implicated in non-urogenital diseases including cancer and coronary artery disease [12;13]. In addition, the detection of differential protein expression in delirium patients may facilitate the understanding of the pathophysiology of disease.

The aim of our present study was to explore whether biomarkers associated with delirium could be detected in urinary protein profiles of hyperactive delirium compared to matched non-delirium ICU- patients.

## Methods

### Patients and delirium assessment

For this explorative study 10 hyperactive delirium post cardiac surgery patients were included and compared with 10 meticulously matched non-delirium patients. For sake of homogeneity, delirium patients after cardiac surgery were included only when they suffered from a hyperactive delirium [2], detected with the validated Dutch version of the confusion assessment method-ICU [14] by well trained ICU nurses [15]. Patients were diagnosed, according to the Peterson criteria [2], as hyperactive delirium when they had only positive RASS during their delirium period. Patients were double checked by a delirium expert (MvdB) for the presence or absence of the delirium to confirm the diagnosis. To secure that only hyperactive delirium patients were included, follow-up took place until patients did not suffer from delirium anymore and only when they had positive RASS scores during their delirium period. In support of the homogeneity of the total group, patients were matched on several important risk factors for the development of delirium [16]. Matching was performed on: gender, age, length of stay on the ICU at the time of urine sample collection, severity of illness score (expressed in Acute Physiology and Chronic Health Evaluation (APACHE)-II score), C-reactive protein (CRP), Aorta clamping time, Euro score, serum and urine creatinine level, modification of diet in renal disease - glomerular filtration rate (MDRD-GFR) and type of operation. Patients suffering from an infection were excluded.

The local Institutional Review Board of Arnhem-Nijmegen (study number 2007/283) indicated that for this study no formal approval was required and no informed consent from patients was needed because of the observational nature of this study and the fact that no additional interventions were carried out. This study was registered on Clinical Trial Register as NCT00604773.

### Sample collection, preparation and measurement

Within 24 hours after the onset of the delirium episode blood and urine were collected for creatinine measurement and urine for proteomics profiling under sterile conditions. As a control, a urine master pool was created according to Vanhoutte et al [13], which consisted of urine of 24 healthy volunteers (age 22-65 years). In brief, first-morning mid-stream urine samples were collected freshly and a master pool reference sample of all healthy volunteers was prepared by mixing together 24 urine samples containing 0.2 mmol creatinine each. Protease inhibitors were added to the urine immediately after the collection and the samples were centrifuged (15 min, 2000*g *at 4°C) and stored in small aliquots at -80°C to minimize freeze-thaw cycles.

### MALDI-TOF-MS analysis: preparation and measurement

To isolate proteins from the urine samples we used magnetic bead (MB) separation [17] with magnetic hydrophobic-interaction chromatography (MB-HIC C8), immobilized metal ion affinity chromatography (MB IMAC-Cu) and weak cation-exchange chromatography (MB WCX) beads. In addition, non-magnetic weak cation-exchange beads (CM10, Bruker Daltronics, Germany) were applied. Urine volume added to the beads was normalized to urine creatinine concentration. A urine volume of maximally 30 μL was used for MB-HIC C8 and HB IMAC-Cu; 15 μL for MB-WCX and 150 μL for CM10 beads. To all samples an internal standard of 5 μL 0.5mM hepcidin 24 was added to normalize peak intensities [18]. MB purifications were performed according to the manufacturer's protocol for serum using the buffers delivered with the kit. For MB-WCX and CM10 beads other buffers were used as described by Kroot [19], based on Park [20]. Pre-treated samples were transferred to a polished steel plate (Bruker Daltronics) and covered with two layers of 5 mg/mL α-cyano-4-hydroxy-cinnamic acid matrix (CHCA; Bruker Daltronics). A linear matrix-associated laser desorption/ionization time-of-flight mass spectrometer (MALDI-TOF MS Microflex, Bruker Daltronics) was used for protein profiling.

### Statistics

Since the exploratory nature of this study, a power calculation for sample size calculation was not performed. Group differences were tested two-tailed using the Mann-Whitney U-test. Mass spectra data obtained after MALDI-TOF MS profiling were analyzed using ClinProt Tools Software (Bruker Daltronics), including univariate statistical analysis and unsupervised hierarchic clustering. A two tailed *P-*value of < 0.05 was considered statistically significant.

## Results

The delirium and non-delirium post-cardiac surgery ICU patients were comparable regarding the matched variables (Table 1). The significantly higher RASS score in the delirium group is a result of the hyperactive delirious state of these patients compared with non-delirium patients. All patients were mechanically ventilated at the time of ICU admission, however, none of the patients was ventilated during the study period. Included patients did not receive any sedatives and all patients were treated with morphine according to our postoperative protocol. All blood and urine was collected in the morning, except for two patients (one in each group) in whom urine was collected in the afternoon.

**Table 1:** Demographic, matched and outcome variables of delirium and non-delirium patients

	Delirium group (N = 10)	Non-delirium group (N = 10)	*p-value*
Admission time (days)	1 [1-1.5]	1 [1]	*0.91*

Gender (Male)	7	6	*0.65*

Age (years)	75 [70-78]	75 [68-78]	*0.73*

RASS-score (median)	0 [0 - 1]	-0.5 [-1 - 0]	*0.007*

APACHE-II score	17 [14-19]	17 [13-21]	*0.88*

C-reactive protein	41 [35-58]	38 [13-48]	*0.28*

Aorta clamping time (minutes)	79 [63-94]	106 [66-115]	*0.35*

Euro score	7 [6-9]	7 [6-12]	*0.70*

Measurement Creatinine after operation in hours	21 [14-43]	21 [15-21]	*0.78*

Serum Creatinine μmol/L	97 [86-114]	86 [57-125]	*0.32*

Urine Creatinine μmol/L	11 [7-6]	8 [6-12]	*0.25*

MDRD-GFR (ml/min/1.73m^2^)	69 [55-75]	71 [52-102]	*0.45*

Type of operation	CABG N = 4	CABG N = 3	*0.87*
	Valve operation N = 2	Valve operation N = 1	
	Valve/CABG N = 2	Valve/CABG N = 3	
	Miscellaneous N = 2	Miscellaneous N = 3	

All values are median [interquartile range 25-75%] unless other reported.

**Figure 1:**
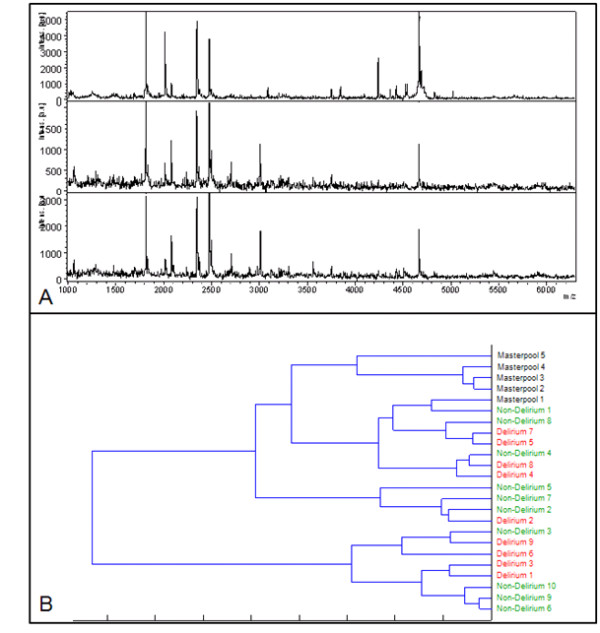
**Protein spectra and hierarchical cluster after profiling with CM10 beads. **A. Protein spectra of masterpool urine (upper panel), a non-delirium patient (middle panel) and a delirium patient (lower panel). The x-axis depicts *m/z *values in Dalton; the y-axis shows the relative peak intensity. B. Unsupervised hierarchical clustering determines whether patient groups can be differentiated solely based on their urine protein profile. On the right hand side the samples are represented. The lengths of the horizontal lines represent the resemblance of the spectra; the shortest lines represent the most alikeness between samples. In this hierarchic cluster our masterpool can be clearly distinguished from the ICU patients, but there is no distinction between delirium and non-delirium patients.

Figure 1 shows representative examples of protein spectra of our master pool urine, which served as a control reference sample, a non-delirium patient and a delirium patient. After unsupervised hierarchic clustering, the urine protein profiles of all ICU patients differed from the master pool urine protein profiles, however, a clear distinction between delirium and non-delirium patients could not be made. Urine proteomics profiling did not reveal protein patterns discriminative for delirium within the ICU patients. However, we found two protein masses to be more abundantly expressed in the non-delirium ICU patients compared to the delirium patients as assessed by the ClinProTools. The clinical relevance of the 11735.7 Da (p < 0.044) mass and its suspected double charged form of 5867.12 Da (p < 0.044) in urine samples of non-delirium ICU patients is, however, questionable since these masses were found in both types of ICU patients and were highly variable. The mean mass intensity and standard deviation of 11735.7 Da in the urine of delirium ICU patients was 22.12 ± 23.47 compared to 32.1 ± 22.1 for the non-delirium ICU patients. For the 5867.12 Da mass this was 12.3 ± 12.3 versus 17.7 ± 10.7, respectively. Efforts to identify these protein masses were not undertaken because of the poor discriminative properties (viz. borderline statistical difference) in delirium ICU patients.

## Discussion

This study shows no relevant differences in urine protein profiles between hyperactive delirium and matched non-delirium post cardiac surgical ICU patients. We could not reproduce the findings from previous studies that reported protein pattern specific for delirium in serum, including haemoglobin-β [8], S100-β [21;22] or other unidentified peaks at *m/z *5030 and 5179 in rats withdrawn from cocaine exposure [10]. This could indicate that no clear hyperactive delirium protein fingerprint is present in the urine of ICU patients or that associated proteins present in brain or serum do not pass the blood-brain-barrier or are not excreted in urine. Although mass spectrometry can be accurately applied to detect proteins over a very wide range with good sensitivity, there are some limitations to biomarker detection using proteomic protein profiling. In this study, beads were used to isolate proteins from urine and to eliminate disturbing salts for MALDI-TOF MS analysis. Disadvantages of this method are that proteins may be lost due to competition for binding to the beads and the use of beads may lead to protein selection. In addition, matrix based ionization is susceptible to signal suppression [23]. Other mass spectrometry methods based on electrospray ionization, such as LC-MS/MS are less susceptible to signal suppression and have a higher sensitivity, but are also more sensitive to interfering compounds such as lipids and detergents. Moreover, LC-MS/MS is time consuming and not suitable for high-throughput screening.

To identify a biomarker pattern specific for a pathological condition it is essential to have homogeneous patients groups. Intra-group variability and the relatively small sample size may have hindered to discover differences between the patient groups. To limit this variability, kidney function and aorta clamping time [24] were meticulously matched between the studied groups. Still, ICU patients have a higher urine protein content as compared to healthy controls (mean 0.22 ± SD 0.13 g/L compared to < 0.100 ± 0.002g/L in masterpool control urine samples), Challenging the discovery of a discriminative protein in these ICU patients a challenge. In addition, the sample size of our study was relatively small, therefore there is a possibility of a type-II error. However, we did not find any clear protein profile difference between delirium and non-delirium patients, which could be an indication of a specific delirium protein in the urine. Consequently we believe that the possibility of a false negative finding is very low.

## Conclusion

No relevant differences in urine protein profiles between hyperactive delirium and matched non-delirium post cardiac surgical ICU was found. MALDI-TOF MS did not reveal a specific hyperactive delirium protein fingerprint in ICU patients.

## Abbreviations

CABG: coronary artery bypass graft; MALDI-TOF MS: Matrix-assisted laser desorption/ionisation-time of flight mass spectrometry; MDRD-GFR: modification of diet in renal disease - glomerular filtration rate; RASS: Richmond Agitation Sedation Scale.

## Authors' contributions

MvdB carried out the study, the statistics and drafted the manuscript. RvS carried out all the proteomics analyses and statistics and drafted the manuscript. SH, PP, RM, JvdH and FR supervised the study and helped to draft the manuscript. All authors read and approved the final manuscript.

## Competing interests

The authors declare that they have no competing interests.
